# Deletion of *Irs2* reduces amyloid deposition and rescues behavioural deficits in APP transgenic mice

**DOI:** 10.1016/j.bbrc.2009.06.032

**Published:** 2009-08-14

**Authors:** Richard Killick, Georgie Scales, Karelle Leroy, Mirsada Causevic, Claudie Hooper, Elaine E. Irvine, Agharul I Choudhury, Laura Drinkwater, Fiona Kerr, Hind Al-Qassab, John Stephenson, Zehra Yilmaz, K. Peter Giese, Jean-Pierre Brion, Dominic J. Withers, Simon Lovestone

**Affiliations:** a King’s College London, MRC Centre for Neurodegenerative Research, Institute of Psychiatry, De Crespigny Park, London, SE5 8AF, UK; b Centre for Diabetes and Endocrinology, University College London, The Rayne Institute, 5 University Street, London, WC1E 6JJ, UK; c Laboratory of Histology, Neuroanatomy and Neuropathology, Université Libre de Bruxelles, Faculté de Médecine, Campus Erasme 808, route de Lennik, Bldg G, 1070 Brussels, Belgium; d Centre for the Cellular Basis of Behaviour, MRC Centre for Neurodegeneration Research, King’s College London, 125 Coldharbour Lane, London, SE5 9NU, UK

**Keywords:** Alzheimer’s disease, Diabetes, Insulin signalling, Tau, APP, Abeta, GSK-3, PP2a, Transthyretin, *Irs2*

## Abstract

As impaired insulin signalling (IIS) is a risk factor for Alzheimer’s disease we crossed mice (*Tg2576*) over-expressing human amyloid precursor protein (APP), with insulin receptor substrate 2 null (*Irs2^−/−^*) mice which develop insulin resistance. The resulting *Tg2576*/*Irs2^−/−^* animals had increased tau phosphorylation but a paradoxical amelioration of Aβ pathology. An increase of the Aβ binding protein transthyretin suggests that increased clearance of Aβ underlies the reduction in plaques. Increased tau phosphorylation correlated with reduced tau-phosphatase PP2A, despite an inhibition of the tau-kinase glycogen synthase kinase-3. Our findings demonstrate that disruption of IIS in *Tg2576* mice has divergent effects on pathological processes—a reduction in aggregated Aβ but an increase in tau phosphorylation. However, as these effects are accompanied by improvement in behavioural deficits, our findings suggest a novel protective effect of disrupting IRS2 signalling in AD which may be a useful therapeutic strategy for this condition.

## Introduction

Alzheimer’s disease (AD) is characterised by extracellular plaques, composed predominantly of β-amyloid (Aβ) peptides and intracellular neurofibrillary tangles (NFTs), composed of hyperphosphorylated forms of the microtubule associated protein tau. The amyloid cascade hypothesis suggests that generation and aggregation of Aβ precedes and indeed promotes tau-related pathology in AD pathogenesis. Altered IIS may be a critical modifier of these processes. For example, IIS regulates both Aβ formation and turnover [1–3] and tau phosphorylation [4,5] while in humans type 2 diabetes and variation in IIS genes are associated with AD [6–8]. Furthermore, in rodent models, brain-insulin resistance increases tau phosphorylation [9] while diet-induced insulin resistance or impaired IGF-1 signalling increases Aβ pathology [10]. Conversely, dietary restriction [11], which enhances insulin action, and IGF-1 therapy [12] ameliorate AD pathology. These observations have led to efforts to develop therapies for AD that improve IIS. However, paradoxically, impaired IIS has also been shown to reduce Aβ aggregation and toxicity in a *Caenorhabditis elegans* model with AD-associated pathology in muscle [13] and impaired IIS is associated with increased longevity in a variety of model organisms [14]. To determine whether toxic or protective effects of reduced IIS upon AD pathology predominate in the mammalian brain, we disrupted IIS in a mouse model of Aβ deposition by crossing plaque-prone *Tg2576* mice with *Irs2^−/−^* mice, a model of type 2 diabetes.

## Materials and methods

### Animals

*Tg2576* mice, expressing the Swedish mutant form of APP (APP—K670N, M671L) [15], were bred into *Irs2^−/−^* mice [16] as follows: male *Tg2576* (C57Bl/6.SJL) were initially crossed with *Irs2^+/−^* females (C57Bl/6). We then bred the F1 *Tg2576* *Irs2^+/−^* males with *Irs2^+/−^* females and the F1 *Tg2576* *Irs2^+/−^* females with *Irs2^+/−^* males. These crosses generated F2 *Tg2576*/*Irs2^−/−^* and WT littermates that were used in all experiments. Mice were maintained on a 12-h light/dark cycle with free access to water and standard mouse chow (4% fat, RM1, Special Diet Services). Mice were handled and all in vivo studies performed in accordance with the United Kingdom Animals (Scientific Procedures) Act (1986) and University College London Ethical Review Process guidelines.

### Protein extraction

Tissue was homogenised at 4 °C in 8-fold (mgs/μL) volume of PBS buffer (10 mM Phosphate, 140 mM NaCl, 2.7 mM KCl, 1 mM EDTA, 10 mM β-glycerolphosphate, 10 mM NaF, pH 7.4, and protease [Roche, UK], phosphatase inhibitor cocktails [Sigma, UK]). Homogenates were spun (100,000*g*, 4 °C for 30 min). The supernatant (S1) was collected and the pellet resuspended with the addition of 1% Triton-X 100, vortexed for 1 min, placed at 4 °C for 10 min and subjected to a repeat centrifugation. The supernatant (S2) was stored at −80 °C. P2 pellets were extracted with a 8-fold volume of 70% formic acid, resuspended, vortexed and placed at 4 °C over night. Following centrifugation at 100,000*g* (4 °C; 30 min), supernatants collected and neutralised with a19 fold volume of neutralisation buffer (1 M Tris base, 0.5 M Na_2_HPO_4_, 0.05% NaN_3_).

### Western blotting

*Phosphorylated tau.* S1 and S2 samples were diluted in equal volumes of 2× reducing sample buffer (BioRad, UK), heated to 100 °C for 5 min, spun and separated on 8% SDS–polyacrylamide gels. Primary antibodies were detected with appropriate secondary antibodies conjugated to fluorophors of 700 or 800 nm, and densitometry performed, using a near infrared Odyssey imager (Licor, UK). Phospho-tau immunoreactivity values were normalized to total tau.

*Detection of full-length APP and APP-CTFs.* S2 fractions were used for detection of full-length holo-APP and COOH-terminal fragments of APP (APP-CTFs). Holo-APP was detected using a 6% Tris–glycine SDS–PAGE with polyclonal anti-APP antibody 369(20). APP-CTFs were detected using 4–12% Bis-Tris NuPAGE system (Invitrogen, UK). Levels of full-length APP were normalized to the levels of actin. APP-CTFβ and APP-CTFα levels were normalized to full-length APP.

*Other proteins.* For all other phospho-proteins, levels were normalized to total protein and non-phosphoproteins to β-actin.

### Immunohistochemistry

*Histological staining*. Brains were dissected between 11 and 14 months of age, fixed in 10% formalin and paraffin embedded. Tissue sections of 10 μm thickness were stained with haematoxylin/eosin, Nissl or Gallyas methods or with Congo red. B19 is a rabbit polyclonal raised to adult bovine tau, reacting with all isoforms in a phosphorylation-independent manner [17]. A rabbit polyclonal antibody to human Aβ_42_ (Bio-Source, Belgium) was used for the detection of Aβ.

*Immunocytochemistry*. Immunohistochemical labelling of brain tissue was performed using the ABC method, as previously described (22). Briefly, tissue sections were treated with H_2_O_2_ and incubated with a blocking solution (10% v/v horse serum in TBS—10 mM Tris, 150 mM NaCl, pH 7.4). After overnight incubation with primary antibody, sections were incubated with horse anti-mouse antibodies conjugated to biotin followed by the ABC complex (Vector Labs, Belgium). Peroxidase activity was revealed using diaminobenzidine as chromogen. For immunolabelling with the Aβ antibodies, rehydrated tissue sections were pre-treated with 100% formic acid for 10 min before incubation with the blocking solution.

*Quantification of Aβ staining*. Digital images of whole brain sagittal sections taken close to the midline, were analysed with the NIH Image J program: the total area covered by Aβ positive deposits was measured using image thresholding and the total cortex surface was measured using manual selection. The area covered by Aβ deposits is expressed relative to the total cortex surface.

### Aβ ELISA

Levels of human Aβ_1–40_ and Aβ_1–42_ in S1 and S2 fractions and formic acid extracted P2 pellets were measured by ELISA according to manufacturers instructions (The Genetics Company, Switzerland).

### Quantitative RT-PCR

Total RNA was extracted from frontal cortex using Triazol according to manufacturers instructions (Sigma, UK). Total RNA (1 mg) was reverse transcribed using random hexamers with a Taqman RT reagent kit (Perkin Elmer, UK). Quantitative RT-PCR for transthyretin was performed as previously described [18] and using Taqman Gene Expression assay FAM/TAMRA primers (Applied Biosystems): *transthyretin* (*Ttr*) (Mm00443267_m1), *Gapdh* (Mm99999915_g1). Primers to mouse IDE, were designed using Universal Probe Library (Roche, UK) software. Real time PCRs were performed on a Lightcycler (Roche, UK) using QuantiTect SYBR green reagent (Qiagen, UK).

### Behaviour

For contextual conditioning mice at 10–12 months were trained in a conditioning chamber (Med Associates, St. Albans, USA) in a soundproof box—after a 120 s introductory period a tone (80 dB, 3.0 kHz) was presented for 30 s, the last 2 s of which coincided with a foot-shock (0.75 mA). A further two tone/foot-shock pairings were administered at 60 s intervals and after a final 60 s period the mice were returned to their home cage. Twenty-four hours after training the mice were re-exposed to the conditioning chamber for 5 min to test for contextual fear memory. Freezing behaviour (defined as complete lack of movement, except for respiration) was scored for 2 s in every 5 s.

## Results

### Expression of mutant APP increases tau phosphorylation in the context of Irs2 deletion

**Fig. 1:**
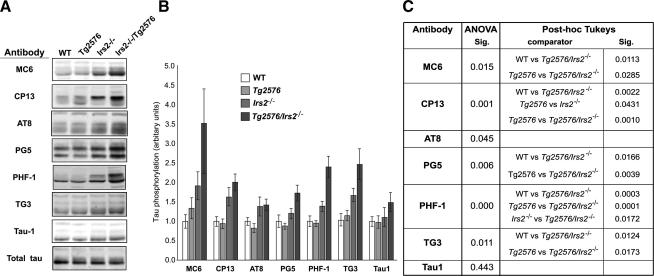
Phospho-tau immunoreactivity is increased in temporal cortex of *Tg2576/Irs2^−/−^* mice. (A) Phosphospecific anti-tau antibodies were used to probe temporal cortex of age-matched wild-type (WT); *Tg2576*; *Irs2*^−^*^/^*^−^ and *Tg2576/Irs2^−/−^* animals. (B) Data shown is normalised to a phosphorylation-independent tau antibody. (C) Analysis by genotype (ANOVA with post hoc Tukeys test; *n *= 29) showed significant increases in tau phosphorylation in *Tg2576/Irs2^−/−^* animals relative to WT and *Tg2576* animals at epitopes recognised by antibodies MC6, CP13, PG5, PHF1, and TG3 but not at the overlapping AT8 and TAU1 epitopes.

**Fig. 2:**
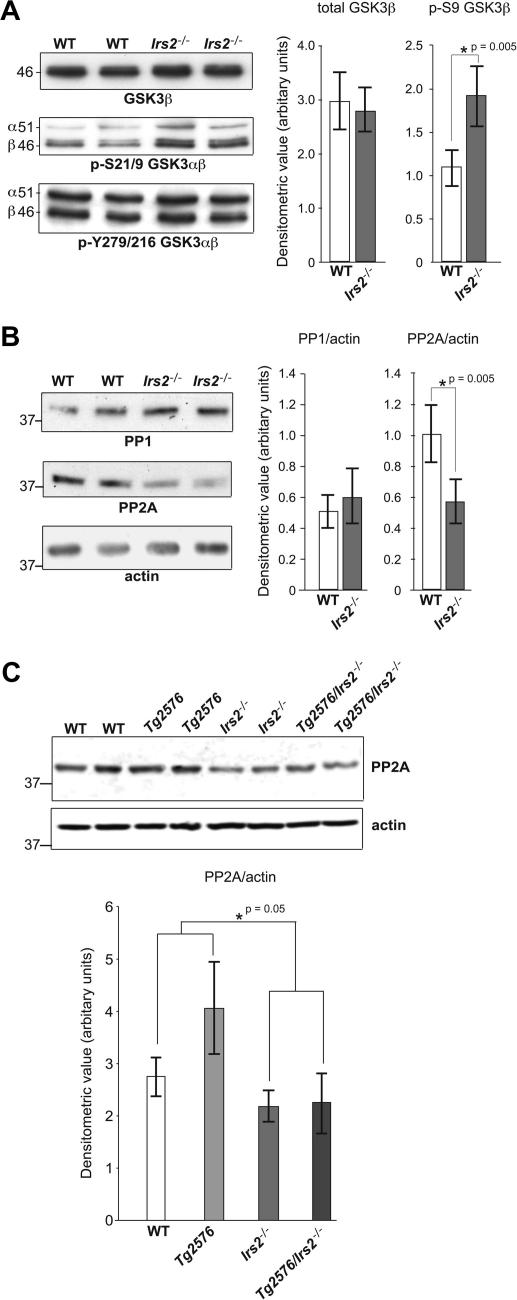
GSK-3 is inhibited and PP2a is reduced in mice lacking *Irs2*. The tau-kinase, GSK-3 and the protein phosphatases PP1 and PP2A were examined in wild-type (WT) and in *IRS2−/−* mice by Western blot. (A) Animals lacking *Irs2* showed no change in total GSK-3 protein or in phosphorylation at the Tyr279 in GSK-3α/GSK-3β 216 epitope. However there was a substantial (*p *< 0.05) increase in GSK-3 phosphorylation at Ser 21 GSK-3α/Ser 9 GSK-3β epitope reflecting relative inhibition of GSK-3 activity in these animals. (B) There was no change in PP1 but a significant increase in PP2A in animals lacking *Irs2.* (C) Comparing PP2a in hippocampus across all four genotypes confirmed a reduction in PP2a in all animals lacking *Irs2* but no effect of APP expression.

Modest increases in tau phosphorylation were seen in 12–15 month-old *Irs2^−/−^* mice. In contrast, in *Tg2576/Irs2^−/−^* mice we detected substantially increased tau phosphorylation indicating that impaired IIS promotes tau phosphorylation which is in turn enhanced by the presence of Aβ pathology (Fig. 1). However, we found no change in tau aggregation (data not shown) but did detect reduced expression of the tau-phosphatase PP2a in animals lacking *Irs2*; this latter observation may, as suggested by others [9], underlie the increased tau phosphorylation seen in *Irs2^−/−^* animals even though the tau-kinase glycogen synthase kinase-3 (GSK-3) was relatively inhibited (Fig. 2).

### Deletion of Irs2 reduces amyloid burden in Tg2576 mice

**Fig. 3:**
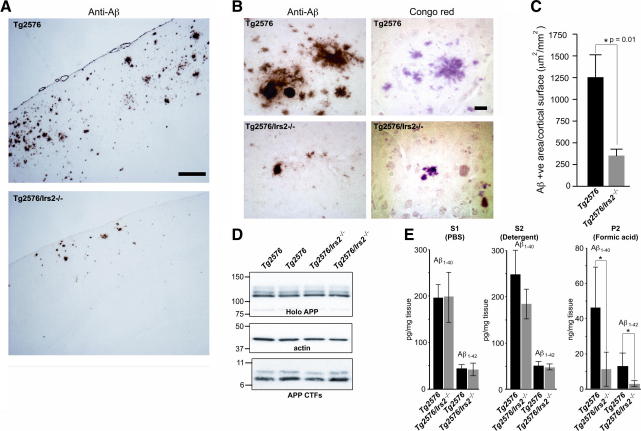
Deletion of *Irs2* reduces Aβ deposition and decreases insoluble *A*β. (A) Immunocytochemical labeling with a human specific anti-Aβ antibody of whole brain frontal cortex sections of 12 month-old *Tg2576* and *Tg2576/Irs2^−/−^* mice. Scale bar = 200 microns. (B) Immunocytochemical with the anti-Aβ antibody (left panels) and histochemical labeling with Congo red (right panels) of temporal cortex sections of 12 month-old *Tg2576* and *Tg2576/Irs2^−/−^* mice, showing the extent of Aβ deposition in representative animals. Scale bars = 50 microns. (C) The mean surface covered by Aβ deposits in the cortex was significantly reduced in *Tg2576/Irs2^−/−^* mice compared with *Tg2576* mice (*n *= 32; ^∗^*p *= 0.01, *t*-test). (D) APP processing was examined by immunoblotting for holo-APP and APP-CTFs in temporal cortex of *Tg2576* and *Tg2576/Irs2^−/−^* animals. Holo-APP values were normlised to actin, CTF values were normalised to holo-APP. No differences were found between genotypes. (E) Human Aβ_1–40_ and Aβ_1–42_ were measured by ELISA in S1 (soluble) and S2 (detergent soluble) fractions and formic acid extracts of P2 pellets from temporal cortex. Significant reductions in Aβ_1–40_ and Aβ_1–42_ were found in formic acid extracts from *Tg2576/Irs2^−/−^* compared to *Tg2576* animals (*n *= 21; ^∗^*p *= 0.0005 [Aβ_1–40_] and 0.002 [Aβ_1–42_], *t*-test).

Twelve month old *Tg2576* mice displayed the expected numerous, large Congophilic Aβ deposits but in contrast, in age-matched *Tg2576*/*Irs2^−/−^* mice plaque number appeared reduced and plaques were smaller and less intensely labelled. Quantification of extracellular Aβ deposits revealed that the area covered by Aβ deposits was significantly reduced in brains of the *Tg2576*/*Irs2^−/−^* mice compared to littermate *Tg2576* animals (*p *= 0.01; Fig. 3).

Measurement of APP metabolites in the temporal cortex of age-matched *Tg2576* and *Tg2576/Irs2^−/−^* mice, showed no differences in holo-APP, or β C-terminal fragments or in soluble Aβ_1–40_ and Aβ_1–42_ levels (Fig. 3). However, there was a significant reduction in insoluble, aggregated Aβ_1–40_ and Aβ_1–42_ levels in *Tg2576/Irs2^−/−^* mice compared to *Tg2576* littermates, closely reflecting the reduction in Aβ load measured by immunocytochemistry. Together these results suggest that altered APP processing does not underlie the alterations in Aβ generation.

### Reduced Aβ burden is associated with increased expression of transthyretin and altered membrane expression of insulin degrading enzyme

**Fig. 4:**
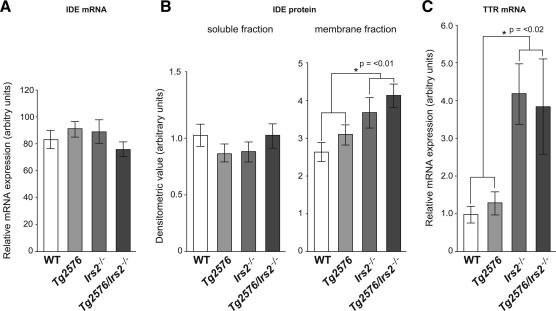
Increase in insulin degrading enzyme and in transthyretin in mice lacking *Irs2*. Amyloid turnover is regulated by proteolysis through proteases such as insulin degrading enzyme (IDE) and by increased clearance following binding to proteins such as transthyretin; both known to be altered in response to insulin signalling. IDE mRNA was unaltered in IRS2−/− mice (A) but protein levels in the membrane-bound fraction were modestly increased (B). Transthyretin mRNA was increased substantially (3.9-fold; *p *< 0.02; SEM 1.05) in both genotypes lacking *Irs2*.

Aβ clearance is regulated by both increased proteolysis and through mechanisms dependent on binding to carrier proteins including transthyretin. Both mechanisms have been implicated in IIS—one of the key Aβ proteases, insulin degrading enzyme (IDE) or insulysin (38), is involved in both insulin and Aβ degradation and transthyretin, recently implicated in Aβ proteolysis as well as Aβ clearance [19], is elevated in both insulin resistant mice and people with type II diabetes [20,21]. We therefore examined the expression of both genes. IDE mRNA expression was not altered but TTR expression was increased 3.9-fold (*p *= 0.01) in animals null for *Irs2* compared to wild-type animals (Fig. 4A,C). We next examined the protein levels of IDE by western blotting. As it has been recently reported that membrane-bound, but not cytosolic, IDE protein is significantly decreased in brain tissue of individuals at high risk of developing AD (40), we examined IDE in both the soluble and detergent soluble fractions. There was a significant increase in membrane associated fraction in *Tg2576/Irs2^−/−^* animals (mean 1 (SD 0.4) vs. 1.6 (SD 0.6); *t*-2.55; *p *< 0.05) (Fig. 4B).

### Deletion of *Irs2* reverses behavioural deficits in *Tg2576* mice

To assess the impact of disrupted *Irs2* on the hippocampal learning and memory ability of the *Tg2576* mice, we tested 10–12 month old mice in contextual fear conditioning, a behavioural paradigm previously shown to be impaired in the *Tg2576* model [22]. One-way ANOVA showed that there was an overall significant difference between the groups (*n *= 32; *F*_3, 28_ = 4.6, *p *= 0.01) due to impairment in *Tg2576* mutants (post hoc Tukey’s *p *< 0.05 for all groups). Thus, the deletion of the *Irs2* gene in the *Tg2576* mice is able to rescue the contextual fear deficit suggesting that the reduction of Aβ load and/or the inhibition of GSK-3 in these animals reversed the effects of over-expression of human APPsw.

## Discussion

Considerable evidence implicates insulin resistance in the pathogenesis of AD and underlies current efforts to treat AD by improving insulin sensitivity. However, we find that disrupting *Irs2* in *Tg2576* mice results in improvement of both Aβ plaque burden and behaviour despite an exacerbation of tau phosphorylation and the presence of insulin resistance. Previously, over-expression of APP in mice was shown to induce transthyretin expression together with evidence of increased IIS [23]. It was suggested that these are protective mechanisms resulting in the absence of the amyloid cascade in mice. Subsequently, and supportive of this hypothesis, transthyretin protein was shown to prevent Aβ toxicity *in vitro* [24,25] and in mice over-expressing APP, neutralisation with antibody or deletion of the TTR gene both enhance pathology [26,27]. In addition to these roles, transthyretin has been shown recently to be, like IDE, an Aβ protease [19] and transthyretin protein levels in CSF are decreased in AD [28,29]. Our data demonstrates a substantial increase in transthyretin expression accompanying the similarly substantial amelioration of plaque pathology, Aβ fibrillisation and behavioural deficits; in line with a protective effect of transthyretin.

In marked contrast to the potentially beneficial reduction in Aβ pathology, we found increased tau phosphorylation in *Tg2276* mice with disruption of *Irs2*. However we found no evidence of tangle formation, consistent with previous observations [9]. The increase in tau phosphorylation was observed at many but not all epitopes examined. The most pronounced changes were at epitopes positioned at the 396/404, and 235 and 231 sites, sites known to be phosphorylated in AD [30]. However, the AT8/TAU1 epitope covering Ser199/Ser202/Thr205, a key GSK-3 site which is also highly phosphorylated in AD brain, was unaffected in the *Tg2576*/*Irs2^−/−^* animals. When we examined tau-kinase activity in *Irs2^−/−^* mice we found no increase in the activities of GSK-3, like others previously [9], or in the GSK3 and CDK5 substrate CRMP-2 (data not shown). These data are consistent with the pattern of tau phosphorylation changes we observed, in particular the absence of an increase in phosphorylation at the key GSK-3 sites—Ser199/Ser202/Thr205. However we did find a highly significant decrease in the tau-phosphatase PP2A in *Irs2^−/−^* mice, suggesting that the mechanism of effect might be mediated not by an increase in kinase activity but by a decrease in the activity of this phosphatase.

The relative role of amyloid versus tau pathologies in influencing neuronal dysfunction and cognitive impairment has been of considerable interest and indeed controversy. The generation of a mouse model with both decreased Aβ aggregation and deposition but increased tau phosphorylation permitted us to directly address this question. Using a standard paradigm of hippocampal dependent learning, contextual fear conditioning, we observed a complete reversal of behavioural deficits in the context of *Irs2* deletion. Interestingly it has been reported that the Aβ induced impairment in LTP, known to be present in the *Tg2576* animals [22], is reversed by insulin [31]. The mechanism whereby insulin might restore LTP is not known but one promising candidate is GSK-3 as we and others have recently demonstrated that GSK-3 inhibition is essential for LTP [32,33]. In the *Tg2576*/*Irs2^−/−^* animals the reduction in Aβ and the relative inhibition of GSK-3 might both, together or separately, contribute to the reversal of the behavioural phenotype.

Although much of the current literature suggests that insulin resistance is an aetiological factor in AD, we have recently demonstrated that mice lacking *Irs1* have increased lifespan and reduced age-related pathology [34] and deletion of *Irs2* in the mouse brain increases longevity [35]. In *C. elegans*, abrogating IIS protects against a range of proteotoxic neuropathologies, including Aβ toxicity [13,36]. Our new findings demonstrate that this is also the case for mammals with specific disruption of *Irs2* and suggests that for therapeutic manipulation of this pathway to be beneficial in the treatment of AD an increased understanding of the complex signalling and gene expression mechanisms downstream of IIS will be required.
